# Neurogenic Lower Urinary Tract Symptoms, Fatigue, and Depression—Are There Correlations in Persons with Multiple Sclerosis?

**DOI:** 10.3390/biomedicines11082193

**Published:** 2023-08-04

**Authors:** Anke K. Jaekel, Julius Watzek, Jörn Nielsen, Anna-Lena Butscher, Pirmin Zöhrer, Franziska Schmitz, Ruth K. M. Kirschner-Hermanns, Stephanie C. Knüpfer

**Affiliations:** 1 Department of Neuro-Urology, Clinic for Urology, University Hospital Bonn, 53127 Bonn, Germany; 2 Department of Neuro-Urology, Johanniter Neurological Rehabilitation Center Godeshoehe GmbH, 53177 Bonn, Germany; 3 Department of Cognitive Rehabilitation, Neurological Rehabilitation Center Godeshoehe GmbH, 53177 Bonn, Germany; 4 Department of Medical Psychology | Neuropsychology and Gender Studies, Center for Neuropsychological Diagnostics and Intervention (CeNDI), Faculty of Medicine and University Hospital Cologne, University of Cologne, 50937 Cologne, Germany

**Keywords:** multiple sclerosis (MS), neuro-urology, neurogenic lower urinary tract symptoms (LUTS), bladder diary (BD), fatigue, depression

## Abstract

The symptoms of multiple sclerosis (MS) frequently include fatigue, depression, and neurogenic lower urinary tract symptoms (LUTS), causing severe burdens on affected individuals. The relationships between these symptoms have not been intensively researched and there are no studies on the detailed influence of the different neurogenic LUTS. We aimed to investigate the relationships between fatigue, depression, and neurogenic LUTS as recorded in bladder diaries by persons with MS. We analyzed the bladder diaries of 274 people and their scores on the Fatigue Scale for Motor and Cognitive Functions and the Centre for Epidemiologic Studies Depression Scale (German version). The neurogenic LUTS were defined as urgency, reduced voided volume, increased standardized voiding frequency, nocturia, and urinary incontinence. Those suffering from incontinence, nocturia, reduced voided volume, or urgency had higher fatigue scores compared to those without these symptoms. Those with nocturia showed significantly higher scores for depression. The severity of urgency and voided volume had the greatest effect on the severity of individuals’ fatigue and depression levels. With increasing urgency, the risk of clinically significant fatigue and depression was expected to increase. Urgency and voided volume correlated most with fatigue and depression. A prospective longitudinal study investigating fatigue/depression after the successful treatment of neurogenic LUTS is needed to clarify causality and offer possible treatment options for fatigue and depression.

## 1. Introduction

In addition to impaired sensory and motor functions, and bladder and bowel dysfunction, persons with multiple sclerosis (MS) suffer from other disease-specific symptoms such as coordination difficulties, visual disturbances, cognitive impairments, and fatigue [1,2]. Fatigue, defined as ‘a sense of exhaustion, lack of energy, or tiredness’, is one of the most common symptoms [3] and is classified as serious, influencing quality of life, daily activities, and workability, regardless of physical disability. Fatigue affects 35–97% of individuals with MS [4]. MS-related fatigue is divided into physical and cognitive fatigue [5]. Physical fatigue results from physical exhaustion due to muscle weakness [6]. Cognitive fatigue can already be present before MS is diagnosed and is defined as a deterioration in cognitive activities resulting from a loss of concentration and memory or emotional instability [4].

Additionally, almost every second person with MS suffers from depression [4]. When fatigue and depression exist side-by-side, they are difficult to differentiate. Together, fatigue and depression affect more than 50% of all persons with MS [4]. There appears to be a strong correlation between both symptoms and shared underlying causes are suspected [7,8]. The primary causes of both conditions are likely to be demyelination, axonal damage, and brain atrophy caused by the inflammatory processes of MS [4]. Secondary causes could include sleep disorders resulting from the mental and physical responses to chronic disease, side effects of medication, or nocturia or other neurogenic lower urinary tract symptoms (LUTS) [1,4].

Up to 80% of persons with MS experience neurogenic LUTS during the course of the disease [9]; these may be the most socially disabling symptoms of the condition [10]. Patients suffer mostly from neurogenic overactive detrusor, followed by detrusor sphincter dyssynergia, bladder hypocontractility, and/or areflexia [10], resulting in urgency, frequency, nocturia, and urinary incontinence or poor bladder emptying [9].

There are significant effects of fatigue and depression on the daily lives of persons with MS and limited availability of causal therapies, especially for MS-related fatigue [11,12,13]. Therefore, it was important for us to thoroughly assess and analyze the potential impact of urinary tract symptoms on these symptoms in order to improve basic knowledge of possible synergistic therapeutical effects. A relationship between fatigue and bladder symptoms has already been assessed in individuals without MS [14,15,16]; however, little is known about the relationship between fatigue and bladder dysfunction in persons with MS [1,17].

Previous studies on this topic have used symptom scores to assess bladder function [1,14,15,16,17]. Using self-reported symptom and quality of life questionnaires is a functional way to assess individual symptoms and provide an overall picture of LUTS [18], but the results are susceptible to unconscious distortion by the patients [19]. Bladder diaries (BDs) can be used alongside symptom scores to provide complementary and corroborating information for an understanding of LUTS [19] and can also be used for therapy control measurement [20]. Therefore, our aim was to investigate the relationships between fatigue, depression, and neurogenic LUTS as objectivated by BDs in persons with MS.

## 2. Patients and Methods

### 2.1. Patients and Assessment

Data for this study were prospectively collected by the Department of Neuro-Urology of an inpatient neurological rehabilitation center and included 274 persons with MS. Data regarding fatigue and depression were collected and provided by the Department of Cognitive Rehabilitation. We analyzed the data retrospectively. Persons aged at least 18 years, who had provided written informed consent and did not have an untreated urinary tract infection were included. The inclusion in the study was independent of the clinical course or severity of the disease. The exclusion criteria were individuals aged <18 years, those who were pregnant or breastfeeding, those with untreated acute lower UTIs, and those who did not provide written informed consent.

We defined neurogenic LUTS as follows: a mean voided volume (VV) of ≤250 mL, a standardized voiding frequency (SVF) of ≥13/d, and the presence of urgency, nocturia, or urinary incontinence (UI). The thresholds were chosen according to a previous analysis [21]. The presence of urgency was captured by anamnesis and the quantification of urgency was captured by BDs. The presence and quantification of the other neurogenic LUTS and the total drinking amount were collected using BDs.

The presence and severity of fatigue were captured by the Fatigue Scale for Motor and Cognitive Functions (FSMC) [5]. The FSMC focuses on motor and cognitive fatigue and contains 20 items (10 for each subscale), which are scored from never (1) to almost always (5). Persons with MS can be categorized as mildly (≥43), moderately (≥53), or severely fatigued (≥63). For the motor subscale the categories are defined as follows: mild (≥22), moderately (≥27), or severely fatigued (≥32). For the cognitive subscale the cut-off values are: mild (≥22), moderately (≥28), or severely fatigued (≥34). Clinically relevant fatigue is defined as a total score of >43 or >22 in the motor or cognitive subgroups [5].

The presence and severity of depression were captured by the Centre for Epidemiologic Studies Depression Scale [22] ((CES-D); in this instance, the German version, Allgemeine Depressionsskala (ADS), was used), which is a 20-item self-rating questionnaire about depressive symptoms [23]. The ADS focuses strongly on cognitive items and less on somatic and fatigue-related items, which helps to avoid measurement problems due to an overlap of depression and fatigue symptoms. The items are answered on a four-point Likert scale from rarely (0) to mostly (3). In the German norm population, a score of >22 indicates clinically relevant depression.

We compared the ADS and the three different FSMC scores (FSMC sum score, FSMC motor, and cognitive subscale) of persons with neurogenic LUTS with those of persons without those symptoms LUTS. The correlations were investigated using three different approaches: firstly, we assessed whether the presence of neurogenic LUTS was associated with higher scores on the FSMC or ADS. An ordinal approach was then used to assess differences in the risk of more severe fatigue between the two groups. Finally, we assessed whether the persons with neurogenic LUTS were more often diagnosed with clinically significant fatigue or depression compared to persons without neurogenic LUTS.

We then correlated the severity of neurogenic LUTS with the ADS and FSMC scores. The severity of neurogenic LUTS was defined by the number of SVF, UI events, nocturia events, and degrees of urgency (measured using a point score from BDs) as well as the number of VVs without any threshold. We analyzed the effect of the severity of neurogenic LUTS using the same three approaches described above.

We investigated the relationship between the number of neurogenic LUTS and the severity of fatigue and depression by correlating the number of neurogenic LUTS with the FSMC and ADS scores.

Finally, we correlated the total drinking amounts (TDAs) from the bladder diaries with the FSMC scores.

This study was conducted in accordance with the Declaration of Helsinki. All patients provided written informed consent. Ethical approval (EK 313/13-University Hospital Bonn) was obtained.

### 2.2. Statistical Analysis

All analyses were performed using the R statistical programming language [24]. For the analysis of Section 3.2.1, the FSMC/ADS scores were distributed along the different symptoms and compared using two-sample *t*-tests. In addition, linear models were calculated with the scores as the target variables and the symptoms, gender, and age of the participants as the influencing variables. In Section 3.2.2, the FSMC cut-off values (no, mild, moderate, severe fatigue) were used to calculate the risk of experiencing mild, moderate, or severe fatigue for the neurogenic LUTS and without neurogenic LUTS groups. An estimation of risks was completed once for each group and category separately and once more using a cumulative logit model considering gender and age, allowing for a statistical comparison of the risks between the groups. The relative risk was calculated to relate the adjusted risks of the symptomatic and asymptomatic persons to each other. An assessment of clinically significant fatigue and depression in persons with and without neurogenic LUTS was completed using logistic regression models for binary outcomes, again adjusting for gender and age (Section 3.2.3). An analysis of the correlation of FSMC and ADS scores with the severity of the neurogenic LUTS (Section 3.3.1) was conducted using the correlation analysis from the previous section, though with a linear regression analysis, with additional adjustments for age and gender.

For consideration of the relative risk of severe or no fatigue (see Section 3.3.2), the effect of the quantitative characteristics of neurogenic LUTS on the severity of fatigue was calculated as a change of 1 SD of neurogenic LUTS in each FSMC scale. The analysis of the binary consideration of clinically significant fatigue/depression (see Section 3.3.3) was again based on logistic regression models, and the analysis of the correlations between the number of neurogenic LUTS and fatigue and depression was based on correlation analyses and linear models. Both analyses were adjusted for gender and age. The correlation of the number of neurogenic LUTS with the FSMC and ADS scores (Section 3.4) was based on simple correlations and regression analysis. The correlation between TDA and fatigue was measured using the Pearson correlation coefficient (Section 3.5). Results with *p*-values < 0.05 were considered statistically significant.

## 3. Results

### 3.1. Patients and Their Disease Characteristics

Our study cohort consisted of 67.2% (184/274) women and 32.8% (90/274) men. The mean Expanded Disability Status Scale (EDSS) score was 4.25 (SD 1.59; min 1, max 8). The mean number of neurogenic LUTS was 2.41 (SD 1.35; min 0, max 5). The mean total drinking amount was 1982.78 mL (SD 686.13; min 110, max 4500). An overview of the frequency of neurogenic LUTS and fatigue and depression in our cohort is provided in Table 1.

The relative risk of urgency was elevated by 1.75 (95% CI 1.17; 2.62, *p* = 0.007) for persons with VVs of ≤250 mL compared to persons with higher VVs and by 1.42 (95% CI 1.04; 1.94, *p* = 0.029) for persons with SVFs of ≥13/d compared to those with lower SVFs.

### 3.2. Fatigue and Depression in Persons with Neurogenic LUTS vs. Persons without Neurogenic LUTS

#### 3.2.1. Assessment of the FSMC and ADS Scores in Persons with and without Neurogenic LUTS

Persons with incontinence and urgency showed significantly higher mean FSMC sum scores compared to those without symptoms. The same symptoms were related to higher scores in the FSMC cognitive subscale. For the FSMC motor subscale, a voided volume of <250 mL had significantly higher mean scores in the analysis. Persons with nocturia had significantly higher mean ADS scores and FSMC cognitive subscale scores. The linear models calculated using the FSMC/ADS scores as the target variables and the symptoms, genders, and ages of the participants as the influencing variables showed the same results. The results are summarized in Table 2.

#### 3.2.2. Assessment of the Risk of Severity of Fatigue in Persons with and without Neurogenic LUTS

We calculated the probability of occurrence of the FSMC severity categories for the groups of symptomatic and asymptomatic persons. Persons with the symptom ‘incontinence’ showed an increased risk for the category ‘severe fatigue’ in the FSMC sum score and had a reduced risk for ‘no fatigue’. The FSMC motor and cognitive subscales pointed in the same direction as the effect estimate, but they did not achieve the precision of the FSMC sum score (Table 3). The results for the symptom ‘VV ≤ 250 mL’ were similar, with a reduced risk for ‘no fatigue’ and an increased risk for ‘severe fatigue’. The symptom ‘SVF’ showed no differences between symptomatic and asymptomatic persons regarding their risk for ‘severe’ or ‘no fatigue’. The symptom ‘nocturia’ had a reduced risk for ‘no fatigue’ for symptomatic persons compared to asymptomatic persons for the FCMC motor subscale. Persons with the symptom ‘urgency’ had an increased risk for ‘severe fatigue’ and a reduced risk for ‘no fatigue’ for all FSMC scales (Table 3).

#### 3.2.3. Assessment of Clinically Significant Fatigue and Depression in Persons with and without Neurogenic LUTS

For the assessment whether persons with neurogenic LUTS had more clinically relevant fatigue and depression than persons without neurogenic LUTS, the cut-off values for ‘no fatigue’ (FSMC score of <43) [5] and ‘no depression’ (ADS score of <22) [23] were used. According to this definition of fatigue and depression, most of the study cohort showed fatigue and approximately half showed depression (Table 4).

Significant differences in the risks for clinically significant fatigue and depression characteristics between symptomatic and asymptomatic persons could not be identified.

### 3.3. Correlation of the Severity of Neurogenic LUTS with Fatigue and Depression

#### 3.3.1. Assessment of FSMC and ADS Scores and Severity of Neurogenic LUTS

Persons with urgency were expected to score 4.98 points higher on the FSMC sum scores if the symptom ‘urgency’ increased by one standard deviation (9.68 points). Analogous results were obtained for the FSCM motor and cognitive subscale calculations and for ADS scores (Table 5).

The opposite was the case for VV, as an increase in the VV was related to a reduction in the severity of the symptom. If the VV increased by one SD (92.75 mL), the FSMC sum score would decrease by 2.98 points and the FSMC motor subscale score would decrease by 1.62 points (Table 5). For the other symptoms (nocturia, SVF, and UI), we could not show these significant correlations.

#### 3.3.2. Assessment of the Risk for Severe Fatigue in Relation to More Severe Neurogenic LUTS

We calculated the potential changes in the risk of more severe fatigue due to a one SD increase in the different neurogenic LUTS. We determined that increasing the VV by one SD (92.75 mL) increased the risk of ‘no fatigue’ by 1.59-fold and reduced that of ‘severe fatigue’ by 0.9-fold according to the FSMC sum score. The motor subscale showed similar results (Table 6). Persons with urgency were expected to have a 0.57-fold decreased risk of ‘no fatigue’ and a 1.08-fold increased risk of ‘severe fatigue’ when the urgency increased by 9.68 points. The FSMC motor and cognitive subscales showed analogous results (Table 6). Changes in the SVF, nocturia, and UI severity did not lead to significant changes in the risk of ‘severe fatigue’.

#### 3.3.3. Assessment of the Risk of Clinically Significant Fatigue and Depression in Persons with More Severe Neurogenic LUTS

Persons with urgency were expected to have a 1.23-fold higher risk for clinically significant depression and a 1.03-fold higher risk for fatigue in the cognitive subscale when urgency was increased by one SD (9.68 points) (Table 7). The other neurogenic LUTS (UI, SVF, VV, and nocturia) showed no significant changes in risk for clinically significant fatigue or depression.

### 3.4. Correlation of the Number of Neurogenic LUTS with Fatigue and Depression

In our cohort, the most common number of different neurogenic LUTS experienced by each person was two (25.4% of the study cohort). A detailed overview is shown in Table 8. The missing data points were caused by the fact that only datasets containing complete information for all five neurogenic LUTS were included.

An analysis based on simple correlations revealed positive effects of the number of symptoms experienced on the FSMC sum score, motor subscale, and ADS. A regression analysis confirmed the same for the FSMC sum score and motor subscale, as well as for the ADS score; in these cases, a higher number of symptoms was associated with higher scores (Table 9).

### 3.5. Correlation between Total Drinking Amount (TDA) and Fatigue

A negative correlation was found between total drinking amount and all FSMC scale scores, but the uncertainty intervals contained both negative and positive values and the *p*-values were quite high. Thus, a significant correlation between these two variables could not be confirmed (Table 10).

### 3.6. Gender-Related Differences between the Correlations of Neurogenic LUTS, Fatigue, and Depression

For the gender-related assessment of the FSMC and ADS scores, we additionally calculated models with separate gender effects and compared this with the non-gender-separated approach. This comparison showed a difference for the symptom ‘SVF ≥ 13/d’ between the genders regarding the effect of the presence of neurogenic LUTS on the FSMC motor subscale (Table A1, Appendix A). For the detailed difference see Table A2, Appendix A The SVF had a significant negative effect on the FSMC motor subscale for males, and we had no significant effect on females. However, the difference between the two effects is significant. Another difference between the genders was presented by the symptom ‘UI’ regarding the effect of the severity of neurogenic LUTS on the FSMC motor subscale (Table A1, Appendix A). For males we had a significant positive effect of UI episodes on the FSMC motor subscale, there was no confirmed effect for women (Table A3, Appendix A). All other neurogenic LUTS did not show gender differences in their effects on FSMC scales or ADS. Similarly, we could not show any gender differences in the additive effect of the individual neurogenic LUTS on FSMC or ADS.

## 4. Discussion

Persons with MS suffer from many different symptoms, such as musculoskeletal and visual impairments, impaired concentration, neurogenic bladder and rectal dysfunction, and fatigue and depression [1,2]. These create significant burdens for the affected persons and for their medical and social care [2], and MS is a major cause of the termination of employment before reaching retirement age [2]. Among the MS symptoms, fatigue and depression create the highest psychosocial burden. Fatigue affects a majority of persons with MS while depression affects approximately half [4].

As fatigue is difficult to treat causally [13], it is important to understand the interactions between fatigue and other MS symptoms as this could lead to new therapeutic approaches. As neuro-urologists, we wanted to identify which of the individual neurogenic LUTS influenced fatigue and depression in which way and to investigate these relationships in more detail. We therefore analyzed the BDs of 274 persons with MS in which the type and severity of their neurogenic LUTS were recorded qualitatively and quantitatively and correlated these with their fatigue and depression scores.

Our data showed that certain neurogenic LUTS were associated with higher fatigue and depression scores. Persons with incontinence, nocturia, and urgency showed significantly higher fatigue sum scores and cognitive subscale scores on the FSMC than those who did not have these symptoms. For the motor subscale, reduced voided volume had a significant association with higher scores in symptomatic versus non-symptomatic persons.

The investigation of the effects of more severe neurogenic LUTS revealed an elevated relative risk of increased scores for urgency and reduced voided volume in the FSMC sum score and motor subscale score. In addition, urgency had these same effects on the fatigue cognitive subscale and depression scores. Furthermore, urgency showed an increased relative risk of clinically significant depression and cognitive fatigue. Urgency proved to be the symptom with the greatest effects on fatigue in both subscales and depression.

Among bladder symptoms, ‘urgency’ is the most difficult to standardize [25]. However, as there was a significant correlation between reduced voided volume, voiding frequency, and urgency in our study, we assumed that, in our study cohort, ‘urgency’ could be understood as the strong desire to void.

The documentation of urgency in the BDs is subject to many influences and it was assessed differently depending on the situation [25,26]. The question of causality therefore arose, especially for this symptom, which is the only one that always shows a correlation to cognitive fatigue besides the correlations with the motor subscale. Fatigue and depression could have a negative influence on the perception of urgency and urgency itself could lead to increased exhaustion (physical and cognitive) and reactive depression. Lin et al. [1] measured interactions between the Actionable Bladder Symptom Screening Tool (ABSST) [27] and fatigue in both directions. However, the sum score for the ABSST did not allow any conclusions to be drawn about a single symptom of impaired bladder function [1], and their study design did not allow them to further clarify the causal relationships.

Ge et al. found interacting associations between fatigue and overactive bladder (OAB) symptoms and a negative impact on psychosocial health in non-neurogenic patients with OAB [14]. However, they were also unable to identify a single bladder symptom that was consistently associated with sleep disturbance/fatigue as the symptomatology was assessed using a questionnaire. For non-neurogenic OAB, Lai et al. [16] found that persons with depression reported more severe incontinence symptoms, greater distress, and poorer quality of life than OAB patients without depression. However, the questionnaires they used to measure OAB symptoms showed no differences between the two patient groups [16]. This suggested that the psychological situation had a reinforcing influence on the symptom experience. The complex interactions between stress, depression, anxiety, and LUTS have been investigated in several other studies [14,15,16,28,29], and, in all cases, causal clarification could not be achieved.

Various authors have, however, considered secondary fatigue to be a consequence of the burden of urinary tract symptoms [1,4,17,30]. The reduced voided volume as an objectifiable item in the bladder diaries, with its multiple correlations, especially with the fatigue motor subscale, appear to support this thesis. No other LUTS showed this stringency in its influence on the motor subscale, and VV had no influence on the cognitive subscale. Muscular exhaustion due to a small micturition volume associated with many sometimes physically challenging micturition procedures would be conceivable. The additive effect of the individual LUTS also shows a correlation with the motor and not with the cognitive subscale and could thus support the thesis of secondary motor fatigue due to LUTS. Lin et al. considered incontinence and nocturia and the resulting lack of sleep to be the causes of fatigue [1]. In our study, however, incontinence and nocturia showed comparatively few effects on the fatigue and depression scores compared to urgency and reduced VV. Ge et al. found in OAB patients, after adjusting their data for nocturia, that nocturia had an impact on sleep disturbance, but not on fatigue [14]. Therefore, the relationship between fatigue and nocturia appeared to have had additional causes other than the postulated nocturia-related sleep disturbance. Again, multidirectional relationships may have been the cause; for example, motor fatigue can lead to incontinence due to muscular weakness and fatigue can contribute to nocturia [30]. However, the effect of fatigue and depression as influencing variables on neurogenic LUTS as the target variables was not the subject of our retrospective study.

The standardized voiding frequency did not show any significant correlation with fatigue and depression, whereas urgency and VV were most strongly correlated with them. Urgency, VV, and voiding frequency are logically related to each other, and we were able to confirm correlations between urgency and reduced VV and increased frequency in our data. A lack of correlation with fatigue or depression could be because of statistical problems due to isolated and very high SVF values but these values were thoroughly verified for correctness. A gender-separated approach to SVF revealed minor differences in the FSMC motor subscale scores, but these were not conclusive. Furthermore, we standardized the voiding frequency for our analysis to 2 l of voided volume per 24 h [31] in order to minimize the influence of a reactive reduction in fluid intake. Due to reduced drinking amount, the excretion volume and, thus, the voiding frequency were lower in reality despite the urge-related lower bladder volume, and the persons were less affected. On the other hand, the reduction in drinking amount could be regarded as a secondary cause of fatigue [17]. Based on our data, the total drinking amount showed a weak negative correlation (according to the Pearson correlation) with fatigue but, in contrast to the results of the study by Cincotta et al., this correlation was not statistically significant [17]. Cincotta et al. [17] investigated a female cohort and examined the hydration status based on urine-specific gravity rather than looking at the total drinking amount. Hydration status depends on many more factors than simply fluid intake. Nevertheless, in looking at our data, the question of why urgency and reduced VV showed multiple correlations with fatigue and depression, but SVF did not, remains unanswered.

We found correlations between most neurogenic LUTS and fatigue and depression. The study design was not intended to clarify causalities but instead to elucidate correlations based on neurogenic LUTS recorded in BDs, which appeared to be more objective than using questionnaires due to their nature as protocols. However, the multiple correlations between fatigue, depression, and neurogenic LUTS, particularly urgency, showed that, although the data on voided volume and frequency in the BDs may have been more objective, the cause of the urgency itself could have been subject to multiple interactions between the bladder and psychosocial factors. Nevertheless, bladder diaries are suitable as a protocol for examining lower urinary tract symptoms in neurogenic and non-neurogenic cases [32,33], and they can help to verify a lack of neurogenic LUTS in persons with MS [34] and to monitor therapy success [20].

In our opinion, two further studies could help to clarify the causality between fatigue, depression, and neurogenic LUTS. First, the urodynamic data from persons with MS should be correlated with fatigue and depression to determine whether both neurogenic LUTS and neurogenic lower urinary tract dysfunction (NLUTD) are correlated with them. Furthermore, a prospective longitudinal study comparing fatigue and depression before and after the successful treatment of neurogenic LUTS is needed and there should also be a focus on gender differences in this context. If a reduction in neurogenic LUTS through therapy measures does not result in a reduction in fatigue and depression, this may either be a coincidence, or urgency, as a central cause of neurogenic LUTS may be triggered by fatigue and depression. If there is an improvement in fatigue and/or depression, this would suggest that the presence of neurogenic LUTS leads to an increase in fatigue and/or depression.

A prospective study would therefore be useful in two ways, as the results could offer the possibility of a new therapeutic approach to fatigue and depression and contribute to clarifying the complex interactions between bladder symptoms and psychosocial aspects.

## 5. Conclusions and Further Directions

We aimed to assess the relationships between neurogenic LUTS and fatigue and depression in a detailed and more objective way by using bladder diaries to capture neurogenic LUTS. We found different significant correlations, voided volume and, in particular, urgency appeared the most frequently, as urgency is the symptom that is the most difficult to objectify. Nevertheless, a bladder diary can be a suitable instrument for recording the quantity and quality of different neurogenic LUTS. To investigate the causality of the interaction between neurogenic LUTS and fatigue and depression, two further studies would be useful: firstly, an investigation of the relationship between the underlying urodynamic changes and fatigue and depression, and secondly, a prospective longitudinal study to investigate the changes in fatigue and depression after successfully treating neurogenic LUTS.

## 6. Limitations

The data were collected by specialized neuro-urological rehabilitation departments, and, thus, the recorded baseline characteristics of the study cohorts may have deviated from those in less specialized settings. The inpatient situation may have influenced the personal stressors of the assessed cohort, resulting in either decreased or increased stress levels compared to those experienced at home. In addition, the people were not distracted by the challenges of daily life and, therefore, they may have been more focused on their MS symptoms. On the other hand, all the participants were exposed to the same external conditions, which were optimized for persons with MS. Another limitation may be the high share of persons suffering from fatigue, which could lead to statistical difficulties. However, this share (94%) corresponded to current data reported in the literature [2,4].

## Figures and Tables

**Table 1:** Frequency of neurogenic lower urinary tract symptoms in the study cohort.

**Neurogenic Lower Urinary Tract Symptom**	**Missing**	**Valid Observations**	**Result**
Urinary incontinence	0	274	81.8% (50/274)
Voided volume of ≤250 mL	88	186	66.7% (124/186)
Standardized voiding frequency of ≥13/d	95	179	25.7% (46/179)
Nocturia	62	212	79.2% (168/212)
Presence of urgency	0	274	47.8% (131/274)
**Fatigue and Depression**	**Missing**	**Valid Observations**	**Result**
FSMC-sum score	61	213	94.8% (202/213)
FSMC-motor subscale	65	209	95.2% (199/209)
FSMC-cognitive subscale	65	209	88.5% (185/209)
ADS	65	209	47.4% (99/209)

FSMC, Fatigue Scale for Motor and Cognitive Functions; ADS, Allgemeine Depressionsskala (the German version of the Centre for Epidemiologic Studies Depression Scale (CES-D)).

**Table 2:** Comparison of the mean FSMC scores and ADS scores of persons with neurogenic LUTS vs. persons without neurogenic LUTS.

*Neurogenicluts*	*FSMC Sum Score*			*FSMC Cognitive Subscale*			*FSMC Motor Subscale*			*ADS*		
	Mean (SD)		*t*-Test (95% CI) *p*-Value	Mean (SD)		*t*-Test (95% CI) *p*-Value	Mean (SD)		*t*-Test (95% CI) *p*-Value	Mean (SD)		*t*-Test (95% CI) *p*-Value
	nLUTS +	nLUTS −		nLUTS +	nLUTS −		nLUTS +	nLUTS −		nLUTS +	nLUTS −	
UI	80.07 (13.9)	74.42 (17.34)	5.66 (0.63; 10.68) 0.028	39.1 (8.59)	35.95 (9.99)	3.15 (0.06; 6.24) 0.046	40.71 (6.87)	38.51 (8.43)	2.2 (−0.31; 4.7) 0.085	24.4 (12.53)	22.01 (11.14)	2.4 (−1.84; 6.64) 0.262
VV ≤ 250 mL	78.49 (13.49)	73.19 (19.63)	5.3 (−0.67; 11.27) 0.081	38.1 (8.13)	36.43 (11.03)	1.67 (−1.78; 5.13) 0.338	40.28 (6.45)	37.1 (9.37)	3.18 (0.28; 6.08) 0.032	24.54 (11.11)	20.74 (11.44)	3.8 (0; 7.6) 0.05
SVF ≥ 13/d	78.98 (14.28)	75.78 (16.44)	3.19 (−2.2; 8.59) 0.242	37.92 (8.45)	37.28 (9.42)	0.65 (−2.55; 3.85) 0.688	40.77 (7.18)	38.71 (7.79)	2.07 (−0.63; 4.77) 0.13	24.89 (11.73)	22.52 (11.23)	2.37 (−1.86; 6.59) 0.267
Nocturia	76.74 (16.82)	71.63 (15.22)	5.12 (−0.78; 11.02) 0.088	37.58 (9.65)	33.94 (9.14)	3.64 (0.04; 7.24) 0.048	39.25 (8.13)	37.36 (7.44)	1.89 (−1.06; 4.84) 0.205	23.91 (11.41)	19.06 (10.28)	4.85 (0.81; 8.88) 0.019
Urgency	78.28 (14.62)	72.95 (18.37)	5.33 (0.86; 9.8) 0.02	38.09 (8.5)	35.17 (10.7)	2.92 (0.3; 5.55) 0.029	40.13 (7.24)	37.85 (8.85)	2.28 (0.08; 4.47) 0.042	23.81 (11.76)	21.23 (11.03)	2.58 (−0.54; 5.69) 0.105

nLUTS, neurogenic lower urinary tract symptoms; UI, urinary incontinence; VV, voided volume; SVF, standardized voiding frequency; FSMC, Fatigue Scale for Motor and Cognitive Functions; ADS, Allgemeine Depressionsskala (the German version of the Centre for Epidemiologic Studies Depression Scale (CES-D)).

**Table 3:** Relative risks for persons with neurogenic LUTS vs. those without neurogenic LUTS regarding the severity of fatigue according to the FSMC classifications [5].

Neurogenic LUTS	FSMC Sum Score		FSMC Motor Subscale		FSMC Cognitive Subscale	
	No (<43)	Severe (≥63)	No (<22)	Severe (≥32)	No (<22)	Severe (≥34)
	Relative risk (95% CI) *p*-value					
UI	0.42 (0.16; 1.10) 0.076	1.19 (1.02; 1.37) 0.024	0.64 (0.31; 1.30) 0.214	1.16 (0.94; 1.44) 0.161	0.47 (0.16; 1.38) 0.169	1.13 (0.98; 1.29) 0.086
VV ≤ 250 mL	0.38 (0.17; 0.84) 0.017	1.24 (1.02; 1.51) 0.035	0.86 (0.43; 1.71) 0.667	1.05 (0.83; 1.34) 0.674	0.29 (0.12; 0.70) 0.006	1.25 (1.04; 1.49) 0.018
SVF ≥ 13/d	0.69 (0.26; 1.84) 0.46	1.07 (0.91; 1.26) 0.421	0.93 (0.44; 1.96) 0.841	1.03 (0.81; 1.30) 0.838	0.53 (0.17; 1.70) 0.29	1.09 (0.95; 1.24) 0.216
Nocturia	0.66 (0.29; 1.47) 0.307	1.11 (0.89; 1.39) 0.355	0.50 (0.26; 0.95) 0.035	1.35 (0.97; 1.87) 0.072	0.69 (0.28; 1.68) 0.409	1.08 (0.89; 1.31) 0.452
Urgency	0.48 (0.25; 0.91) 0.024	1.19 (1.03; 1.39) 0.022	0.59 (0.35; 1.01) 0.052	1.22 (1.00; 1.48) 0.05	0.48 (0.24; 1.00) 0.05	1.14 (1.00; 1.31) 0.045

UI, urinary incontinence; VV, voided volume; SVF, standardized voiding frequency; FSMC, Fatigue Scale for Motor and Cognitive Functions; CI, confidence interval.

**Table 4:** Frequency of clinically significant fatigue and depression in the study cohort.

N = 274	Missing % (N)	No Significant Fatigue % of Valid Observations (N)	Significant Fatigue % of Valid Observations (N)
FSMC sum score	22.3 (61)	5.2 (11)	94.8 (202)
FSMC motor subscale	23.7 (65)	4.8 (10)	95.2% (199)
FSMC cognitive subscale	23.7 (65)	11.5 (24)	88.5 (185)
		**No Significant Depression** **% of Valid Observations (N)**	**Significant Depression** **% of Valid Observations (N)**
ADS	23.7 (65)	52.5 (110)	47.4 (99)

FSMC, Fatigue Scale for Motor and Cognitive Functions; ADS, Allgemeine Depressionsskala (the German version of the Centre for Epidemiologic Studies Depression Scale (CES-D)).

**Table 5:** Calculated expected changes in the FSMC and ADS scores due to changes in the severity of neurogenic LUTS.

Neurogenic LUTS	Mean (SD)	FSMC Sum Score	FSMC Motor Subscale	FSMC Cognitive Subscale	ADS
		Change in FSMC and ADS scores due to a 1 SD increase in neurogenic LUTS (95% CI) *p*-value			
UI episodes (number)	0.87 (1.93)	1.47 (−1.06; 4) 0.254	0.55 (−0.7; 1.8) 0.387	0.41 (−1.04; 1.87) 0.575	0.12 (−1.7; 1.94) 0.894
VV (mL)	224.36 (92.75)	−2.98 (−5.48; −0.49) 0.02	−1.62 (−2.83; −0.4) 0.009	−1.07 (−2.54; 0.41) 0.155	−1.64 (−3.44; 0.17) 0.075
SVF (number)	11.14 (8.01)	1.15 (−1.42; 3.72) 0.379	0.83 (−0.42; 2.08) 0.193	0.07 (−1.44; 1.57) 0.932	−0.36 (−2.2; 1.49) 0.702
Nocturia episodes (number)	0.13 (0.45)	0.91 (−1.64; 3.46) 0.483	0.69 (−0.57; 1.95) 0.282	0.13 (−1.34; 1.59) 0.864	0.66 (−1.17; 2.49) 0.476
Severity of urgency (points)	11.54 (9.68)	4.98 (2.52; 7.45) <0.001	2.26 (1.02; 3.49) <0.001	2.48 (1.05; 3.92) <0.001	2.84 (1.04; 4.64) 0.002

UI, urinary incontinence; VV, voided volume; SVF, standardized voiding frequency; FSMC, Fatigue Scale for Motor and Cognitive Functions; SD, standard deviation; CI, confidence interval; ADS, Allgemeine Depressionsskala (the German version of the Centre for Epidemiologic Studies Depression Scale (CES-D)).

**Table 6:** Relative risk for persons regarding the severity of fatigue according to the FSMC classifications [5] in relation to changes in neurogenic LUTS severity.

Neuroegnic LUTS		FSMC Sum Score		FSMC Motor Subscale		FSMC Cognitive Subscale	
		No (<43)	Severe (≥63)	No (<22)	Severe (≥32)	No (<22)	Severe (≥34)
	Mean (SD)	Change in risk for fatigue expression by a 1 SD increase in neurogenic LUTS Relative risk (95% CI) *p*-value					
VV (mL)	224.36 (92.75)	1.59 (1.06; 2.39) 0.024	0.90 (0.81; 1.00) 0.049	1.80 (1.15; 2.80) 0.01	0.90 (0.81; 0.99) 0.027	1.17 (0.85; 1.63) 0.338	0.94 (0.84; 1.07) 0.366
Severity of urgency (points)	11.54 (9.68)	0.57 (0.34; 0.94) 0.028	1.08 (1.03; 1.13) 0.002	0.58 (0.33; 0.99) 0.047	1.06 (1.02; 1.11) 0.006	0.50 (0.32; 0.78) 0.002	1.16 (1.09; 1.24) <0.001

VV, voided volume; FSMC, Fatigue Scale for Motor and Cognitive Functions; SD, standard deviation; CI, confidence interval.

**Table 7:** Relative risk for persons regarding clinically significant fatigue and depression due to changes in the severity of neurogenic LUTS.

		FSMC Sum Score	FSMC Motor Subscale	FSMC Cognitive Subscale	ADS
	Mean (SD)	Relative risk for clinically significant fatigue/depression with an increase in neurogenic LUTS by 1 SD (95% CI) *p*-value			
Severity of urgency (points)	11.54 (9.68)	1.01 (1; 1.03) 0.096	1.01 (1; 1.03) 0.099	1.03 (1; 1.06) 0.034	1.23 (1.05; 1.43) 0.008

SD, standard deviation; CI, confidence interval; ADS, Allgemeine Depressionsskala (the German version of the Centre for Epidemiologic Studies Depression Scale (CES-D)); FSMC, Fatigue Scale for Motor and Cognitive Functions.

**Table 8:** Frequency of number of neurogenic LUTS in the study cohort.

		**Number of Neurogenic LUTS**					
	Missing	0	1	2	3	4	5
Number of persons	97	12	39	45	36	36	9
Percent	35.4%	6.8%	22%	25.4%	20.3%	20.3%	5.1%

**Table 9:** Correlation of number of neurogenic LUTS with FSMC and ADS scores.

	FSMC Sum Score	FSMC Motor Subscale	FSMC Cognitive Subscale	ADS
N	153	150	150	151
Mean number of neurogenic LUTS (SD)	2.43 (1.36)	2.44 (1.35)	2.44 (1.35)	2.44 (1.36)
Adjusted mean FSMC and ADS scores (95% CI)	76.38 (73.76; 79.00)	39.11 (37.84; 40.38)	37.23 (35.69; 38.77)	22.38 (20.50; 24.27)
Correlation (95% CI) *p*-value	0.22 (0.07; 0.37) 0.006	0.24 (0.09; 0.39) 0.003	0.16 (0; 0.31) 0.05	0.21 (0.05; 0.36) 0.009
Change in FSMC and ADS score by one additional neurogenic LUTS (95% CI) *p*-value	2.45 (0.58; 4.33) 0.011	1.28 (0.36; 2.2) 0.007	0.92 (−0.2; 2.03) 0.106	1.56 (0.23; 2.89) 0.022

N, number of valid observations; SD, standard deviation; CI, confidence interval; neurogenic LUTS, lower urinary tract symptoms; ADS, Allgemeine Depressionsskala (the German version of the Centre for Epidemiologic Studies Depression Scale (CES-D)); FSMC, Fatigue Scale for Motor and Cognitive Functions.

**Table 10:** Correlation between total drinking amount (TDA) and FSMC score.

	FSMC Sum Score	FSMC Motor Subscale	FSMC Cognitive Subscale
N	176	173	173
Mean FSMC score (SD)	75.53 (16.84)	38.94 (8.18)	36.56 (9.8)
Mean TDA value (SD)	1982.78 (686.13)	1982.78 (686.13)	1982.78 (686.13)
Pearson correlation (95% CI) *p*-value	−0.12 (−0.27; 0.02) 0.098	−0.12 (−0.26; 0.03) 0.123	−0.11 (−0.26; 0.04) 0.144

SD, standard deviation; FSMC, Fatigue Scale for Motor and Cognitive Functions; CI, confidence interval.

## Data Availability

The data presented in this study are available on request from the corresponding author. The data are not publicly available due to privacy.

## Appendix A

**Table A1:** Comparison of the gender-separated correlation between neurogenic LUTS and FSMC and ADS scores.

Neurogenic LUTS	FSMC Sum Score	FSMC Motor Subscale	FSMC Cognitive Subscale	ADS
	Comparison of the gender-separated correlation between the presence of neurogenic LUTS and FSMC and ADS scores (*p*-value)			
UI	0.748	0.410	0.814	0.925
VV ≤ 250 mL	0.575	0.361	0.870	0.292
SVF ≥ 13/d	0.410	0.034	0.760	0.342
Nocturia	0.103	0.125	0.164	0.242
Urgency	0.781	0.534	0.369	0.601
	Comparison of the gender-separated correlation between the severity of neurogenic LUTS and FSMC and ADS scores (*p*-value)			
UI episodes (number)	0.321	0.038	0.711	0.089
VV (mL)	0.938	0.431	0.672	0.514
SVF (number)	0.483	0.884	0.402	0.849
Nocturia episodes (number)	0.585	0.742	0.480	0.559
Severity of urgency (points)	0.680	0.525	0.718	0.366

UI, urinary incontinence; VV, voided volume; SVF, standardized voiding frequency; FSMC, Fatigue Scale for Motor and Cognitive Functions; ADS, Allgemeine Depressionsskala (the German version of the Centre for Epidemiologic Studies Depression Scale (CES-D)).

**Table A2:** Comparison of the mean FSMC motor subscale scores of persons with neurogenic LUTS vs. persons without neurogenic LUTS separated by gender.

*Neurogenic LUTS*	*Gender*	*FSMC Motor Subscale*				
		Neurogenic LUTS +		Neurogenic LUTS −		*t*-Test (95% CI) *p*-Value
		N	Mean (SD)	N	Mean (SD)	
SVF ≥ 13/d	male	33	41.82 (6.9)	66	38.45 (8.29)	−3.46 (−6.62; −0.30) 0.032
	female	7	35.86 (6.82)	46	39.07 (7.09)	3.92 (−2.12; 9.96) 0.202

SVF, standardized voiding frequency; FSMC, Fatigue Scale for Motor and Cognitive Functions; SD, standard deviation; CI, confidence interval.

**Table A3:** Calculated expected changes in the FSMC motor subscale scores due to changes in the severity of neurogenic LUTS separated by gender.

Neurogenic LUTS	Gender	UI Episoedes Mean (SD)	FSMC Motor Subscale Adj. Mean (95% CI)	Change in FSMC Motor Subscale Scores Due to a 1 SD Increase in Neurogenic LUTS (95% CI) *p*-Value
UI Episodes (number)	male	0.78 (1.56)	80.41 (42.88; 117.94)	1.06 (0.08; 2.04) 0.033
	female	0.97 (2.46)	26.05 (−7.26; 59.37)	−0.32 (−1.19; 0.55) 0.463

UI, urinary incontinence; FSMC, Fatigue Scale for Motor and Cognitive Functions; SD, standard deviation; CI, confidence interval.
